# A Novel Benthic Phage Infecting *Shewanella* with Strong Replication Ability

**DOI:** 10.3390/v11111081

**Published:** 2019-11-19

**Authors:** Zengmeng Wang, Jiulong Zhao, Long Wang, Chengcheng Li, Jianhui Liu, Lihua Zhang, Yongyu Zhang

**Affiliations:** 1 Key Laboratory of Biofuels, Shandong Provincial Key Laboratory of Energy Genetics, Qingdao Institute of Bioenergy and Bioprocess Technology, Chinese Academy of Sciences, Qingdao 266101, China; wangzm@qibebt.ac.cn (Z.W.); zhaojl@qibebt.ac.cn (J.Z.); wanglong@qibebt.ac.cn (L.W.); licc@qibebt.ac.cn (C.L.); 2 University of Chinese Academy of Sciences, Beijing 100049, China; 3 CAS Key Lab of Separation Sciences for Analytical Chemistry, National Chromatographic Research and Analysis Center, Dalian Institute of Chemical Physics, Chinese Academy of Sciences, Dalian 116023, China; jianhuil1810@dicp.ac.cn (J.L.); lihuazhang@dicp.ac.cn (L.Z.)

**Keywords:** coastal sediments, benthic phage, *Shewanella*, phage genome, phylogenetic analysis

## Abstract

The coastal sediments were considered to contain diverse phages playing important roles in driving biogeochemical cycles based on genetic analysis. However, till now, benthic phages in coastal sediments were very rarely isolated, which largely limits our understanding of their biological characteristics. Here, we describe a novel lytic phage (named *Shewanella* phage S0112) isolated from the coastal sediments of the Yellow Sea infecting a sediment bacterium of the genus *Shewanella*. The phage has a very high replication capability, with the burst size of ca. 1170 phage particles per infected cell, which is 5–10 times higher than that of most phages isolated before. Meanwhile, the latent period of this phage is relatively longer, which might ensure adequate time for phage replication. The phage has a double-stranded DNA genome comprising 62,286 bp with 102 ORFs, ca. 60% of which are functionally unknown. The expression products of 16 ORF genes, mainly structural proteins, were identified by LC-MS/MS analysis. Besides the general DNA metabolism and structure assembly genes in the phage genome, there is a cluster of auxiliary metabolic genes that may be involved in 7-cyano-7-deazaguanine (preQ_0_) biosynthesis. Meanwhile, a pyrophosphohydrolase (MazG) gene being considered as a regulator of programmed cell death or involving in host stringer responses is inserted in this gene cluster. Comparative genomic and phylogenetic analysis both revealed a great novelty of phage S0112. This study represents the first report of a benthic phage infecting *Shewanella*, which also sheds light on the phage–host interactions in coastal sediments.

## 1. Introduction

In their natural environment, phages are important agents influencing the physiology and survival of prokaryotes and have significant impacts on the microbially driven biogeochemical cycles [1,2,3]. In recent decades, extensive studies on marine phages in seawater have been carried out via field investigation, laboratory isolation, gene-based analysis, and metaproteomic methods. Now we have a certain understanding of the biological characteristics, community composition, and ecological functions of the planktonic phages. The total amount of bacteriophage in the ocean is estimated to be ca. 10^30^, about 15-fold times than prokaryotes [2]. Phage infections significantly control the bacterial mortality and, through viral shunt, affect nutrient cycling and food web dynamics [2,4]. Recently, the most abundant plankton in the oceans, SAR11 bacteria, formerly considered immune to phage predation, was found also susceptible to dominant phages (i.e., pelagiphages) in seawater [5]. Meanwhile, phage infection can promote the coevolution of bacteria and phages [4]. Many phages have been found to contain host genes via lateral gene transfer and participate in the host metabolism, such as the genes (*psbA* and *psbD*) encoding for photosynthetically important proteins, which were recently found in a number of cultured cyanophage genomes [6]. By contrast, so far there is still a great lack of knowledge about benthic phages, particularly the benthic phages in coastal sediments. For example, although previous gene-based analysis has revealed that the coastal sediments contain extremely diverse phages and may play important roles in driving carbon cycles [7,8], benthic phages have been rarely isolated from coastal sediments in the laboratory, which largely limits our understanding of their biological characteristics. At present, isolation and characterization of benthic phages infecting ecologically important bacteria is especially vital for us to explore the mystery of phage activities in coastal sediments.

*Shewanella* is an ecologically important bacterial genus, whose members are widely distributed in freshwater, seawater, sediment, and deep ocean [9]. Members of the genus *Shewanella* have multiple metabolic capabilities and have potentials in bioremediation and microbial energy generation [10]. Meanwhile, they were considered to be model organisms to study the microbially driven carbon-cycling process [11]. Till now, only nine *Shewanella* phage genomes have been sequenced, including four siphoviruses (i.e., 1/44, 3/49, Spp001, and SppYZU05) and five mycoviruses (i.e., 1/4, 1/40, 1/41, SFcil, and SppYZU01) [12,13,14,15]. The nine *Shewanella* phages were isolated from various environments, but none was from marine sediments. Phage SppYZU01 infecting *S. baltica* and phages SppYZU05, Spp001 infecting *S. putrefaciens* were isolated from waste effluents of a seafood market [12,13]. Phage SFCi1 infecting *S. fidelis* was isolated from the seawater of Mission Bay [14]. The remaining five *Shewanella* phages were isolated from Baltic Sea ice [15]. As we know, members of *Shewanella* are widespread in marine sediments [9] and play important roles in extracellular electron transfer [16], reduction of humic acids and iron [10], and degradation of persistent organic compounds, such as petroleum, chlorinated solvents, tiazine, and tetrachloroethene [17]. However, phages infecting *Shewanella* spp. from marine sediments had never been reported. Even sediment phages are rarely reported. So far, to the best of our knowledge, only two phages isolated from coastal sediments have been comprehensively identified (including the genome annotation), whose hosts are *Vibrio parahaemolyticus* and *Acinetobacter baumannii*, respectively [18,19].

In this study, *Shewanella indica* KJW27, which was isolated from coastal sediments of the Arabian Sea [20], was used as the host bacterium, to isolate its phages from the coastal sediments of Yellow Sea, China. Via biological identification and genome analysis, we found that certain unique characteristics of this benthic phage might be related to phage life strategy and environmental adaptability in coastal sediments. The isolation and characterization of this phage open a window for future explorations of the interactions between phage and the ecologically important *Shewanella* spp. in marine sediments.

## 2. Materials and Methods

### 2.1. Isolation and Purification of Benthic Phages

The bacterial strain used in this study was *S. indica* KJW27, which was isolated in 2011 from the coastal sediments of Arabian Sea, India [20]. The strain was grown on RO medium (yeast extract 1 g·L^−1^, peptone 1 g·L^−1^, sodium acetate 1 g·L^−1^, artificial seawater 1 L, pH 7.8–8.0), at 30 °C, in a shaking incubator. Coastal sediment samples (5 g) were collected from Aoshan Bay of the Yellow Sea, China (120°79′ N; 36°44′ E), in September 2018, and immediately transferred into a 100 mL of RO culture medium, for 7 days, in order to promote phage reproduction and increase the probability of phage isolation. Then, the filtrate containing phage particles were collected by filtration through 0.22 µm pore-size filters (Millipore, Bedford, MA, USA). One milliliter of the phage filtrate was added to *S. indica* KJW27 culture (100 mL) and incubated for 24 h, at 30 °C, in a shaker. The culture was filtered again and serially diluted to determine phage activity via the double-layer agar method [21]. Single phage plaques were picked up from the plate by using a sterile pipette and then purified five times, using the double-layer agar method. The purified phages were cultivated for expansion in liquid RO medium. The mixture was centrifuged at 10,000× *g* at 4 °C, for 15 min, to collect the phage-containing supernatant. Supernatants were collected after filtration through 0.22 μm pore-size filters, in order to remove the remaining cells and debris. Phage lysates were then treated with 2 ng·L^−1^ of DNase I and 2 ng·L^−1^ RNase A, at room temperature, for 1 h. After digestion, NaCl was added to the treated lysates (final concentration: 1 M) and incubated at 4 °C for 1 h. The treated lysates were centrifuged at 10,000× *g* for 10 min at 4 °C. The supernatant was filtered through a 0.22 µm pore-size filter to remove the debris. The filtrate was concentrated by polyethylene glycol 8000 precipitation (final concentration: 100 g·L^−1^) overnight, at 4 °C. The mixture was centrifuged at 10,000× *g*, for 60 min, at 4 °C, and the precipitates were resuspended in 6 mL of SM buffer (100 mM of NaCl, 8 mM of MgSO_4_, 50 mM of Tris-HCl, at pH 7.5). Afterward, the phages were purified by CsCl gradient ultra-centrifugation (gradient-density: 1.5 g·mL^−1^, 200,000× *g*, 8 h, 4 °C; CP-100WX, Hitachi Limited, Tokyo, Japan). The purified phage particles were collected and dialyzed three times in SM buffer, using 30 kDa super-filters (UFC5030, Millipore), and they were then used for morphologic observation and phage genome extraction.

### 2.2. Phage Morphological Observation Using Transmission Electron Microscopy (TEM)

The CsCl-purified phages were prepared for imaging on a 200-mesh copper grid, using a negative stain with 2% aqueous uranyl acetate, as described previously [22]. Samples were viewed at 80 kV voltage, using an H-7650 transmission electron microscope (Hitachi Limited, Tokyo, Japan). Images were taken using GATAN INC CCD image transmission system (Gatan Inc., Pleasanton, CA, USA).

### 2.3. Detection of Phage Host Range

The host range of phage S0112 was tested by the spot test method with the ability to form plaques on bacterial lawn culture. Besides *S. indica* KJW27, the tested bacteria included seven strains from marine sediments, i.e., *Alginatibacterium sediminis* ALS 81, *Woeseia oceani* SDUM189001, *Sediminicola luteus* SDUM 701001, *Kordiimonas sediminis* N39, *Shewanella chilikensis* JC5, *Shewanella basaltis* CJW-54, and *Shewanella japonica* KCTC22435; ten strains from seawater or saline water, i.e., *Marinobacter vinifirmus* D7035, *Halomonas denitrificans* D7027, *Vibrio alginolyticus* CIP 82.01, *Vibrio neocaledonicus* NC 470, *Shewanella algae* JCM 21037, *Vibrio azureus* NBRC 104587, *Vibrio harveyi* NBRC 15634, *Vibrio parahaemolyticus* NBRC 12711, *Vibrio campbellii* CAIM 519, and *Roseobacter denitrificans* OCH114. Briefly, 2 µL of phage suspension (~10^6^ phage particles) was spotted onto the surface of double-layer agar plate inoculated with the tested strains at 30 °C, for two weeks, and plaque formation was examined each day. Three replicates were tested for each bacterial strain.

### 2.4. Lipid Detection in the Viral Capsid

To determine whether there were lipids in the viral capsid, 1 mL of phage suspension was mixed with 0, 20, and 200 µL of pure chloroform, vigorously shaken for 1 min, and then kept at room temperature for 30 min. The samples were centrifuged at a low speed to ensure that the phages were kept in the supernatant. The supernatant containing phages were dropped onto an *S. indica* KJW27 plate, in order to observe the emergence of plaques [23].

### 2.5. The Influence of the External Factors on Phage Particles Stability

To investigate the effect of external factors on the stability of the phage particles, the following external factors were tested: pH (2, 4, 10, and 12), temperature (−20, 15, 20, 28, 30, 37, 40, 50, 60, 95, and 100 °C), detergents (0.1% CTAB, 0.09% SDS, and 0.1% Sarkosyl), and organic solvents (63% ethanol, 90% acetone, 50% DMSO, and chloroform). The effect of osmotic shock on phage particles production was also analyzed, as previously described [24]. 

### 2.6. Lysis Profile Assay

Host culture was inoculated with phage lysate at a multiplicity of infection (MOI) of 0.01. Their density was monitored by OD_600_ measurement at the 30 min intervals up to 8 h. The bacterial density (OD_600_), survival of host bacteria after phage infection (CFU·mL^−1^) and lysate titer (PFU·mL^−1^) were analyzed during the experiment. To investigate the number of surviving cells after phage infection, 100 μL samples were harvested at times indicated above, and serial dilutions (10-fold each) were made in RO medium. Then, 50 μL of each dilution was spread onto RO agar plates. The CFU of each sample was calculated by counting the colonies. To estimate the lysate titer (PFU·mL^−1^), 100 μL samples were removed every 30 min, and their serial 10-fold dilutions were prepared in SM buffer. Then, the PFU of each dilution was calculated by counting the plaques on the bacterial lawn.

### 2.7. One-Step Growth Curve

One-step growth curve experiments were performed, as previously described [25]. Briefly, phages were added to 1 mL aliquots of the bacterial culture at mid-exponential growth phase at MOI = 0.01. After 20 min, cells were centrifuged at 6000× *g* for 10 min at 4 °C, to remove the non-adsorbed phages in the supernatant, and the pellets were resuspended in 1 mL of RO medium. This process was repeated twice. Then, 50 µL of the resuspended culture (the bacteria and adsorbed phages) was transferred to 50 mL of RO medium and incubated over 8 h at 30 °C [26]. Two sets of duplicate samples were taken every 15 min, for eight hours, and chloroform (1% final concentration) was added to the second set, in order to release the intracellular phage. The two samples were serially diluted (10-fold each) and immediately plated for phage titration, using the double-layer agar plate method [27]. Three replicates of this experiment were carried out in this study.

To investigate whether the phage replication was independent of the host, 50 mL of the host culture was added, with rifampicin, at various concentration, including 0, 5, 10, and 20 μg·mL^−1^ (final concentration) [28]. Twenty min after addition of the rifampicin, the phage lysate was added to the host culture at MOI of 1.0 and incubated for 20 h at 30 °C. The phage titer was assayed as described above.

### 2.8. Phage Genome Extraction

The CsCl-purified phages were incubated with proteinase K (100 mg·mL^−1^), SDS (100 μg·mL^−1^), and EDTA (0.5 mol·L^−1^, pH 8.0) and incubated at 55 °C, in water, for 3 h. The digested sample was extracted by using an equal volume of phenol/chloroform/isoamyl alcohol (25:24:1). The samples were completely mixed before centrifugation at 12,000× *g* and 4 °C for 5 min. The aqueous phase was purified by adding chloroform/isoamyl alcohol (24:1) before centrifugation at 12,000× *g* and 4 °C for 10 min. This step was repeated twice. The aqueous phase was mixed with isopropyl alcohol and incubated overnight at −20 °C, before centrifugation at 12,000× *g* for 15 min. The precipitate was washed twice with cold ethanol (70%) and then resuspended in 100 μL of TE buffer (10 mM of Tris-HCl, 1 mM of EDTA, pH 8.0). The purified phage genomic DNA was stored at −20 °C before analysis.

### 2.9. Genomic Analysis

Sequencing libraries were constructed by using TruSeq DNA Library Prep Kits (Illumina, San Diego, CA, USA). Whole genome was sequenced by using the Illumina Hiseq X Ten (2 × 150 bp), and the sequencing data reached coverage of 50,000× and were assembled by using SPAdes genome assembler (v3.12.0), with default k-mer lengths. Open reading frames (ORFs) were predicted with the GeneMarkS online server (http://exon.gatech.edu/Genemark/genemarks.cgi) and were then verified manually, using the NCBI ORF Finder online server (http://www.ncbi/nlm.nih.gov/orffinder/). The putative function of translated products was analyzed and annotated, using BLAST searches against the NCBI non-redundant (nr) protein database with E-value ≤ 10^−3^. A termini analysis and PhageTerm were used to identify the phage’s termini and genome packaging [29,30], while tRNAscan-SE was used to scan tRNA sequences [31]. Progressive Mauve algorithm was used to compare the phage S0112 with the other phages infecting *Shewanella* spp. isolated from different environments and phage VpKK5 isolated from coastal sediments [32]. Gene maps were created by using the Java Operon combined with the genome annotations. To test any spacers of CRISPR array within the phage genome, the phage genome was searched against viral spacer database of the Integrated Microbial Genome/Virus (IMG/VR) database (https://img.jgi.doe.gov/cgi-bin/vr/main.cgi) [33], as well as in spacers CRISPRs loci of its host *S. indica* KJW27, the isolates used in this study [34].

### 2.10. Proteomic Analysis

Proteins of phage S0112 were prepared according to the filter-aided sample preparation procedure [35]. All samples were analyzed on a Dionex UltiMate 3000 nanoLC System (Thermo Fisher Scientific, Waltham, MA, USA) coupled online to an LTQ Orbitrap Velos mass spectrometer (Thermo Fisher Scientific, Waltham, MA, USA). The data were acquired in a data-dependent mode. The obtained spectrometry information was searched against the database created from translated open reading frames that have been found in the genome of phage S0112 by using Mascot 2.3.02 software (Matrix Science Inc., Boston, MA, USA). The important parameter settings for protein identification were as follows: peptide mass tolerance = 20 ppm, MS/MS tolerance = 0.05 Da, enzyme = trypsin, missed cleavage = 2, and fixed modification: Carbamidomethyl (C), and variable modification (M).

### 2.11. Construction of Phylogenetic Tree

The amino acid sequences of DNA polymerase subunit B of phage S0112 was aligned with those of the closely related bacteriophages, deposited in the NCBI database, using MUSCLE implanted in the MEGA 7.0 [36]. The maximum likelihood phylogenetic tree with 1000 bootstrap replications for DNA polymerase subunit B amino acid sequences was constructed by the model of Poisson. To find the closest relatives of phage S0112, the ViPTree was used to construct the viral proteomic tree [37]. 

### 2.12. Genome Recruitment

In order to evaluate the similarity between the phage S0112 genome and the virioplankton communities, the sequences of the phage ORFs and the databases of the Pacific Ocean Virome (POV), Global Ocean Survey (GOS) were compared by using reciprocal best-hit BLAST [5,38,39]. Samples in POV and GOS were collected from aquatic environments during various seasons, at multiple depths, and from different habitats. In addition, to completely compare whole genome rather than only the ORF, we search for the phage S0112 genome in the IMG/VR database, which is the largest available database related to viral genomics, including the genome of cultivated viruses, prophages derived from cultivated microbial isolates, and viral fragments generated by the metagenomes [33]. 

### 2.13. Nucleotide Sequence Accession Number

The genome sequence of the phage S0112 is available under accession number MK675901. The raw reads were submitted to the SRA and can be retrieved via SRA PRJNA573990.

## 3. Results and Discussion

### 3.1. Phage Basic Characteristics: Infection Mode, Morphology, Host Ranges, and Existence or Absence of Lipids in Phage Capsid

**Figure 1:**
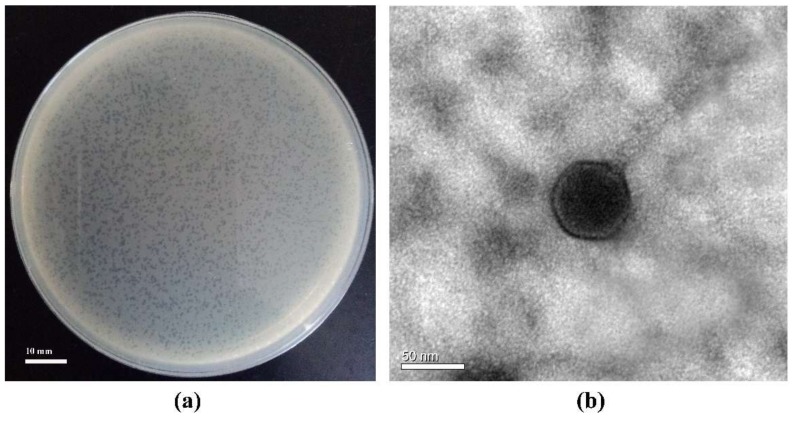
(**a**) Phage plaques formed in double-layer agar plates and (**b**) transmission electron microscopy image of the phage S0112.

The phage S0112 is a lytic phage infecting *S. indica* KJW27, which can form small clear round plaques on plates with an average diameter of 0.5–1 mm after two days of incubation with bacterial hosts (Figure 1a). Morphological analysis via TEM showed that phage S0112 had a polyhedral capsid (62 ± 3 nm in diameter) and a long, flexible, non-contractile tail (97 ± 1 nm in length and 10 ± 1 nm in width) (Figure 1b). 

Host range analysis revealed that phage S0112 could not infect any other bacterium except *S. indica* KJW27 among the eighteen tested strains, which include four different *Shewanella* spp. from marine sediments and seawater that have similar evolutionary status with *S. indica* KJW27 (Table 1). After being treated with chloroform, the phage can still survive on a bacterial lawn, indicating the absence of lipids in phage capsid. As introduced above, only two phages (i.e., VpKK5 and AB1) isolated from coastal sediments have been identified comprehensively in the past [18,19]. Based on the few reports on coastal sediment phages, the three known benthic phages including phage S0112 in this study were found to affect only very limited species within a specific genus. However, we believe that the benthic phages in coastal sediments must have much more complex and diverse biological characteristics than what we know now and remain to be explored. Isolating more phages from coastal sediments is of great significance to discover their largely unknown characteristics. 

### 3.2. Lysis Profile Assay and Sensitivity of Virions to Physical and Chemical Factors

**Figure 2:**
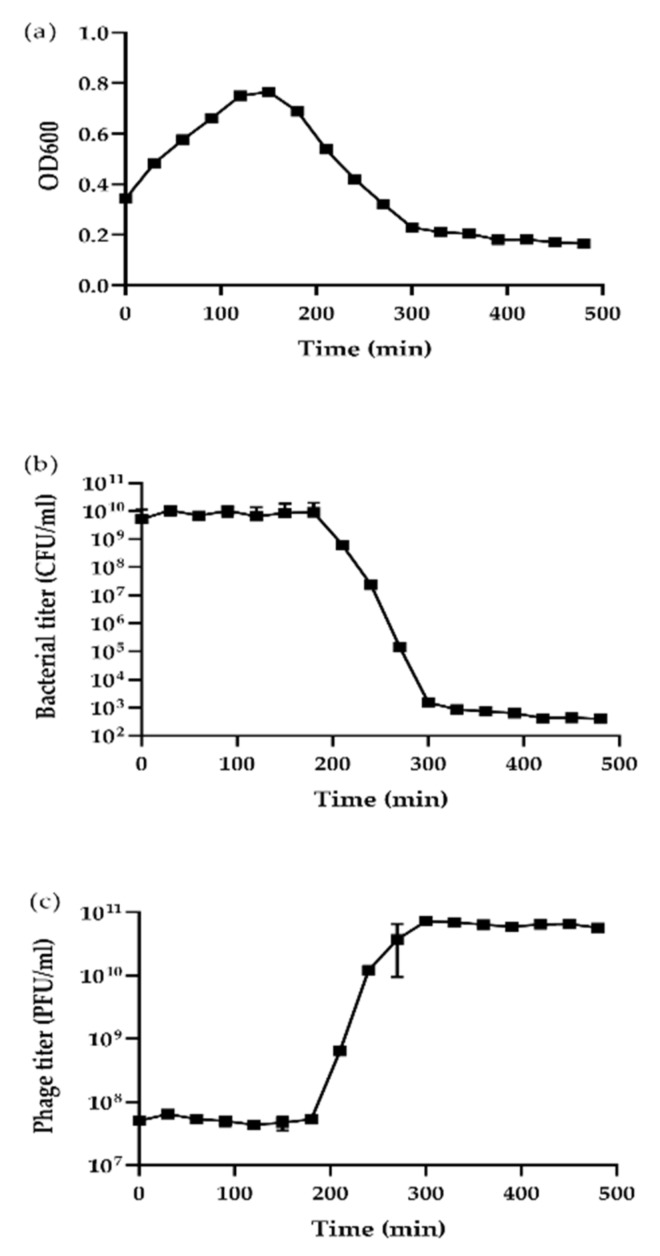
Kinetics of lytic development of *Shewanella* phage S0112 in *Shewanella indica*. Results are shown as (**a**) bacterial culture density measured at OD_600_, (**b**) the number of host cells after phage S0112 infection per 1 mL (CFU·mL^−1^), and (**c**) the number of phages per 1 mL (PFU·mL^−1^). Error bars show standard deviations among triplicate samples.

The entire course of the lysis of host bacteria spends almost five hours (Figure 2). Meanwhile, we found that phage S0112 could infect *S. indica* KJW27 and form clear plaques on plates within a pH range of 4–12, temperature from −20 to 60 °C, at the presence of organic solvents (63% ethanol, 90% acetone, 50% DMSO, and chloroform), detergents (0.1% Sarkosyl and 0.1% CTAB), and osmotic shock, whereas when the pH value was lower than 2, or the temperature was higher than 95 °C, or there was 0.09% SDS, the phage infection activity disappeared (Table 2).

### 3.3. High Replication Capability Reflected by the High Burst Size

**Figure 3:**
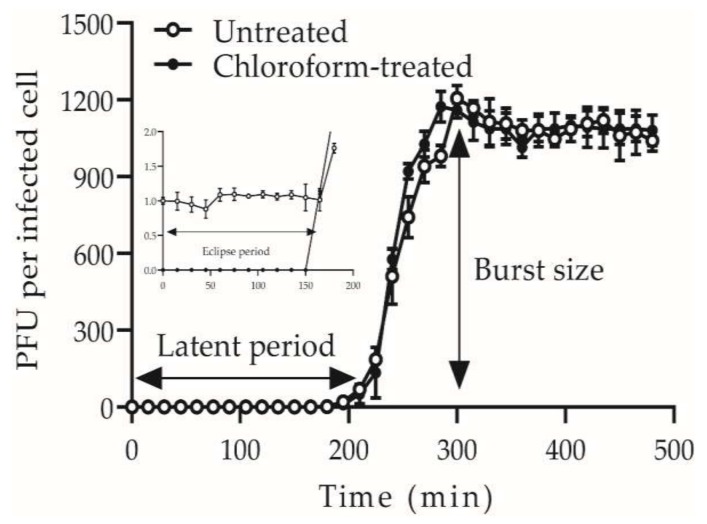
One-step growth curve of *Shewanella* phage S0112. Open circles represent non-chloroform-treated samples; closed circles represent chloroform-treated samples. Error bars show standard deviations among triplicate samples.

**Figure 4:**
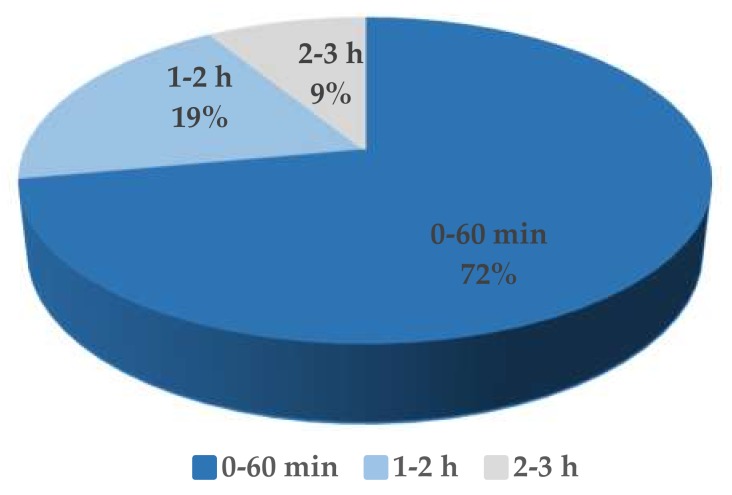
The latent period of sixty-eight phages infecting *Roseobacter* spp., *Vibrio* spp., *Pseudoalteromonas* spp., *Aeromonas* spp., *Acinetobacter* spp., *Escherichia* spp., etc.

The one-step growth curve of the phage S0112 revealed that its eclipse and latent period of phage infection occurred during 2 h 45 min and 3 h post-infection, respectively (Figure 3), which is much longer than most phages isolated before (Figure 4). We randomly counted sixty-eight phages isolated from aquatic environments infecting strains such as *Roseobacter* spp., *Vibrio* spp., *Pseudomonas* spp., *Aeromonas* spp., *Acinetobacter* spp., *Escherichia* spp., etc., and found that 91% of the viruses have the latent period of less than 2 h (Table S1). Comparatively, the latent periods of the phages which were isolated from seawater or sewage and have similar hosts with that of phage S0112 (i.e., *Shewanella* spp. strains) are only 12.7–60 min [12,13,14]. We speculate that the physiological state or doubling time of bacterial hosts may vary in different environments, which possibly have influences on the phage latent period. The hosts listed in Table S1., such as *Vibrio* spp., *Escherichia* spp., and *Acinetobacter* spp., usually have a rapid doubling time of <30 min, whereas the doubling time of the phage S0112′s host *Shewanella indica* KJW27 is about 70 min. At present, it is not clear about the potential relationship between the host doubling time and the phage latent period, which is worth exploring in the future.

Concurrent with the long phage latency, the burst size of phage S0112 was as high as 1170 phage particles per infected cell, which is 5–10 times higher than that of most phages. It has been suggested that the increase of latent period might result in the formation of more progeny phages, i.e., higher burst size [40]. Based on this, it is not abnormal for phage S0112 to have such a high burst size, because the longer latent period can provide ample time for the replication and packaging of progeny phages. Moreover, the host metabolic activity and its survival environment were considered to be closely related to phage latency and burst size [41]. Fast-growing cells under favorable environments can produce more progeny phages than in tough environments [41]. In this study, phage S0112 and its host were all isolated from nutrient-replete coastal sediments. High content of organic matter in coastal surface sediments can boost bacterial growth. When vigorous bacteria are infected by phages, their stronger metabolic activities might provide more energy for viral replication. This is likely why phage release or phage burst size is often higher in laboratory culture than in natural environment. Based on the very limited reports of laboratory-cultured benthic phages, the other two coastal sediment phages, i.e., VpKK5 and AB1, infecting *Vibrio parahaemolyticus* and *Acinetobacter baumannii* respectively, also showed relatively higher burst sizes, which is 180 and 409 phage particles per infected cell [18,19]. 

In addition, viral production in marine sediments was reported to be higher than in the water column [42]. Although the nutrient-rich coastal sediments are beneficial for bacterial growth, the complexity of sediment components, such as the presence of abundant extracellular enzymes, may be detrimental to phages and promote phage decay and degradation [42]. Therefore, we propose that the strong replication ability of benthic phages might be a survival strategy adapting to the coastal sediment environment to ensure that enough progeny phages can survive the tough conditions. Meanwhile, the fluidity of phages in sediments is relatively weaker than that in water columns, which may lead to a lower contact rate between phages and their hosts. The benthic phages with high burst size can produce more progeny phages, to ensure the adequate occurrence of phage–host contacts and maintain their own survival via infections. 

To test if the phage S0112 replication is independent of host, we analyzed the sensitivity of phage infection to rifampicin, which can inhibit host RNA polymerase (RNAP) by binding to the *β* subunits [28]. The addition of 5 μg·mL^−1^ of rifampicin to host culture at prior (20 min) infection completely abolished the production of phage progeny, suggesting that phage S0112 cannot complete the infection cycle independently without host RNAP (Table S2).

### 3.4. Genomic Features of the Phage S0112

The phage S0112 genome consisted of 62,286 bp of linear ds DNA, with the average G+C content of 44.7%, which is lower than that of its host (51.2%) [20]. PhageTerm analysis showed that the DNA termini and packaging mechanisms of phage S0112 is similar with that of T5-like phages. No tRNA genes were found in the phage genome. Furthermore, the genome of phage S0112 was searched against viral spacer database of IMG/VR and spacers within CRISPRs of its host. The result showed no match between phage S0112 and viral spacers sequences within CRISPRs. The phage genome was predicted to encode 102 putative open reading frames (ORFs), of which only 42 ORFs were assigned putative functions based on their amino acid sequence homology to known proteins. About 59% of the ORFs (60 ORFs) are functionally unknown, among which 21 ORFs were annotated as hypothetical proteins and 39 ORFs had no any homology with protein databases (Table S3). Among the 42 ORFs with known functions, eight ORFs shared similarities with that of *Vibrio* phage VpKK5, which is also a siphovirus isolated from the marine sediments [18], with percent identities of 42–59%; six ORFs share similarities with the *Pantoea* phage vB_PagS_Vid5 and *Pseudomonas* phage MP1412; and eight ORFs shared low identity with *Alphaproteobacteria* phage Jl001, Roseophage RDJL Ф 1 and *Burkholderia* phage BcepGomr. All the genes with homology to S0112 are from siphoviruses. 

**Figure 5:**
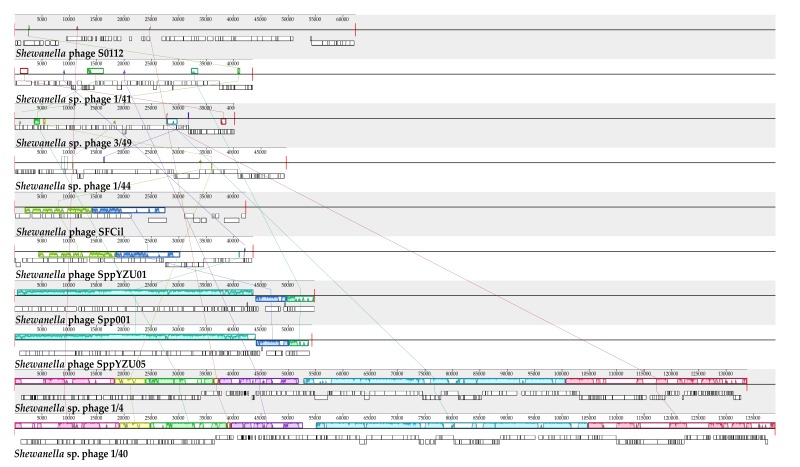
Multiple genome alignments of the whole genomes of all *Shewanella* phages, using Mauve algorithm. The *Shewanella* phages in the analysis are isolated from different environments, including Baltic Sea ice, seawater, sewage, waste effluents from fish market, and coastal sediments. Colored boxes indicate homologous DNA regions between genomes without genomic rearrangements. Genomic similarity is represented by the height of the bars, which corresponds to the average level of conservation in that region of the genome sequence. Completely white regions represent fragments that were not aligned or contained sequence elements specific to a particular genome.

So far, nine *Shewanella* phage genomes were reported [12,13,14,15]. These phages vary significantly in phage size, GC content, protein amount, and isolation sources (Table 3). Multiple genome alignments of the nine *Shewanella* phages and phage S0112 showed that the genome of phage S0112 has a very small amount of similarity with others [32] (Figure 5). Among phage S0112 genome, only three ORFs of phage S0112 show certain similarities with other *Shewanella* phages, including S0112_005 (DNA/RNA helicase), S0112_018 (primase/helicase), and S0112_053 (hypothetical protein).

**Figure 6:**
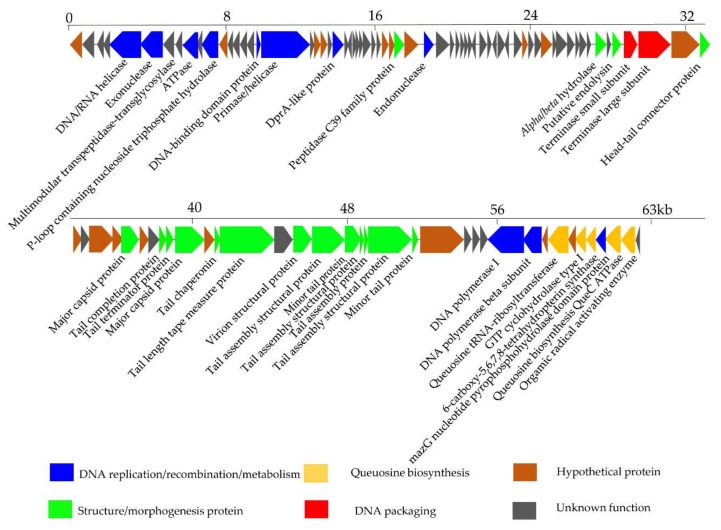
Genomic map of phage S0112. The phage S0112 genome is presented schematically with ORFs indicated by leftward or rightward oriented arrows, based on the direction of transcription. Gene features (DNA packaging, structure/morphogenesis protein, DNA replication/recombination/metabolism, and queuosine biosynthesis) are color-coded according to the legend below the figure.

The encoded proteins of phage S0112 can be divided into four functional modules: DNA packaging module, structure/morphogenesis module, DNA replication/recombination/metabolism module, and queuosine biosynthesis module (Figure 6). The DNA packaging module mainly includes the gene product S0112_063 (terminase small subunit) and S0112_064 (terminase large subunit, sharing 49.3% amino acid identity with the corresponding proteins of *Fodinicurvate sediminis*). It is reported that terminase generally consists of one larger submit and one small submit. The role of the terminase small submit is to specifically recognize the packaging initiation site (*cos* or *pac*) and helps assemble a holoenzyme complex with a larger submit which can cleave genomic DNA at special site, to ensure the whole genome into preassembled empty capsids, using its endonuclease and ATPase activities [43].

The structure/morphogenesis module includes head–tail connector protein (S0112_066), major capsid protein (S0112_071 and _076), tail completion protein (S0112_074), tail terminator protein (S0112_075), tail chaperonin (S0112_078), tail length tape measure protein (S0112_079), virion structural protein (S0112_081), tail assembly structural protein (S0112_082, _084, _085, and _086), minor tail protein (S0112_083 and _087), peptidase C39 family protein (S0112_034), *alpha*/*beta* hydrolase (S0112_060), and putative endolysin (S0112_062). Among this module, the tape measure protein usually exists in siphoviruses and is involved in the assembly of phage tails and the determination of the tail length [44]. Endolysin is a lytic enzyme used to lyse the cell wall of the host. The endolysin consists of two domains: One is the N-terminal domain, which is responsible for degrading bacterial peptidoglycan, and the other is the C-terminal domain, called holin, which is mainly bound to the cell membrane [45]. 

The DNA replication/recombination/metabolism module in phage genome includes ten ORFs, whose gene products include DNA polymerase I (S0112_092), DNA polymerase subunit B (S0112_093), DNA/RNA helicase (S0112_005), primase/helicase (S0112_018), DNA-binding domain protein (S0112_017), exonuclease (S0112_006), endonuclease (S0112_036), ATPase (S0112_009), P-loop containing nucleoside triphosphate hydrolase (S0112_011), and DprA-like protein (S0112_023). Among them, DNA polymerase is vital for phage DNA replication, and its coding gene is relatively conservative in many tailed phages [46]. Primase and helicase found in the genome of phage S0112 are of key importance for DNA replication. The primosome formed by primase and helicase can synthesize a short RNA primer, which can be elongated by DNA polymerase [47]. Exonucleases are enzymes that participate in the process of breaking phosphodiester bonds at either 3′ or 5′ end [48]. Other enzymes including endonuclease, DNA-binging protein, and DprA-like protein play important roles in DNA repair and transcription regulation. It is worth noting that ATPase encoded by ORF 9 is similar to the ATPase of the benthic *Vibrio* phage VpKK5, which was also isolated from coastal sediments. ATPase is important for DNA replication, DNA recombination, and DNA packaging [18].

Interestingly, we found ORF 99 in the phage genome of S0112 is an auxiliary metabolic gene encoding the MazG nucleotide pyrophosphohydrolase domain [49]. MazG was implicated as a regulator of programmed cell death in *Escherichia coli* by interfering with the MazEF toxin–antitoxin system through reducing the intracellular guanosine 3′,5′-bispyrophosphate (ppGpp) levels. The small nucleotide ppGpp, as a global regulator of gene expression, whose accumulation can cause the reorientation of bacterial transcription, and only genes important for starvation survival can be expressed [50,51]. Given the role of MazG in *E. coli*, it was proposed that phages containing *mazG* gene may trick the host to mimic a nutrient replete cell state by reducing the accumulation of ppGpp in the host cells, so that phages can utilize the host RNA transcription system to amplify the progeny phages [52]. However, a recent study found that the purified MazG from cyanophage has no binding or hydrolysis activity to ppGpp [49]. Instead, cyanophage encoded MazG protein can bind and hydrolyze dGTP and dCTP deoxyribonucleotides, and it was speculated that it may indirectly regulate the host stringent response by hydrolyzing other nucleotide substrates [49]. Here, the specific function of MazG gene in the phage S0112 remains to be studied.

Another feature of phage S0112 is the presence of a gene cluster consisting of five functional genes encoding QueC (7-cyano-7-deazaguanine synthase), QueD (6-carboxy-5,6,7,8-tetrahydropterin synthase), FolE (GTP cyclohydrolase I), queuosine tRNA-ribosyltransferase, and organic radical activating enzyme. This kind of gene cluster has also been observed in other phages, such as *Rhizobium* phage RHEph04 [53], R5C [54], *Vibrio* phage VpKK5 [18], *Escherichia coli* phage 9g [55], and the genus *Seuratvirus* [56]. This gene cluster involves in the biosynthesis of a precursor, i.e., 7-cyano-7-deazaguanine (preQ_0_), of queuosine or archaeosine [57]. Queuosine is a well-known nucleoside derivative that modifies tRNAs by replacement of guanine at certain position [58]. Queuosine modification in bacteria was considered a way to improve reading frame maintenance [59], but this opinion is still controversial, as a recent study did not observe this function in *Escherichia coli* [60]. Meanwhile, a phage infecting *Escherichia coli* that contains homologues of queuosine biosynthesis genes, including organic radical activating enzyme, queuosine tRNA-ribosyltransferase, QueC, QueD, FolE, and (GAT)-QueC (glutamine amidotransferase class-II) was found to be resistant to a wide range of restriction endonucleases, by modifying its DNA (converting deoxyguanosine to 2′-dexoy-archaeosine) [55,56,57]. As reported, phages belonging to the genus *Seuratvirus* contain QueC, QueD, and QueE, as well as FolE, modify their DNA with queuosine, as they lack homologues of (GAT)-QueC, which is required for the insertion of archaeosine [56]. Our analysis showed that phage S0112 contains only QueC homologues without the homologues of (GAT)-QueC, and we propose that phage S0112 may modify their DNA in a similar manner to the genus *Seuratvirus*. Further work is needed to verify the biological role of the queuosine biosynthesis genes in phage S0112.

### 3.5. Mass-Spectrometric Identification of Phage Proteins

LC–MS/MS analysis identified 16 out of the 102 predicted ORF expression products, among which, eleven are phage structural proteins, including major capsid protein, minor tail protein, tail length tape measure protein, tail assembly structure protein, tail terminator protein, head-tail connector protein, and virion structural protein (Table 4). Another five proteins (encoded by ORF1, 65, 68, 69, and 80), having no significant homologs in the database, were classified as uncharacterized proteins. However, other predicted ORF gene products, including the functional proteins, were not detected via LC–MS/MS analysis. The reason might be that the expression level of these proteins is relatively low and did not reach the detection limit of LC–MS/MS in our experiment. 

### 3.6. Phylogenetic Analysis

**Figure 7:**
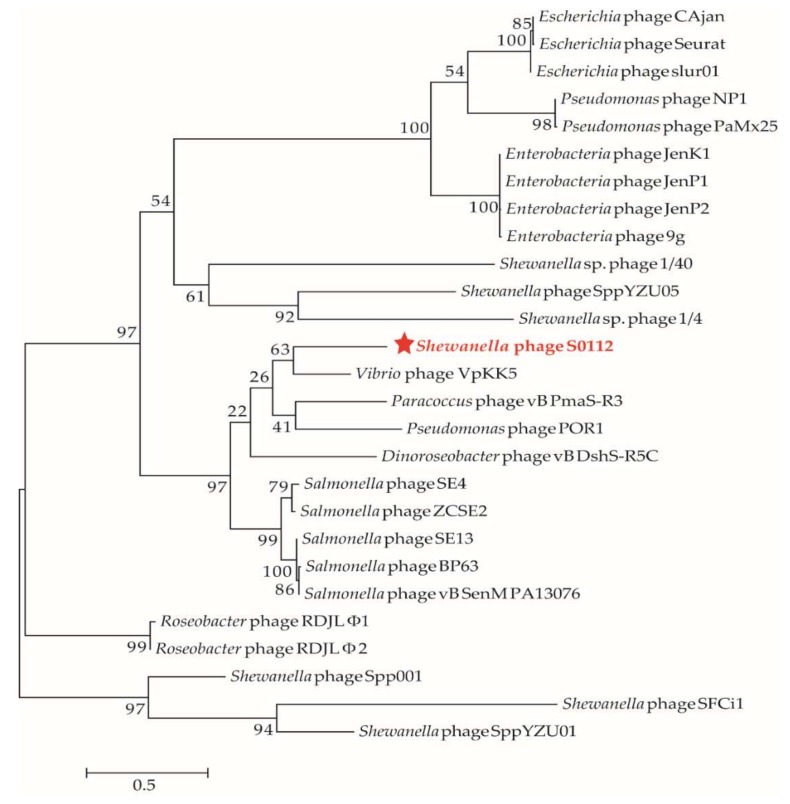
The maximum likelihood phylogenetic tree of phages based on their DNA polymerase subunit B amino acid sequences. The red star represents *Shewanella* phage S0112 isolated in this study. The reference sequences were collected from the NCBI database. The tree was constructed based on the MUSCLE alignment of MEGA 7.0. The bootstrap values were based on 1000 replicates.

**Figure 8:**
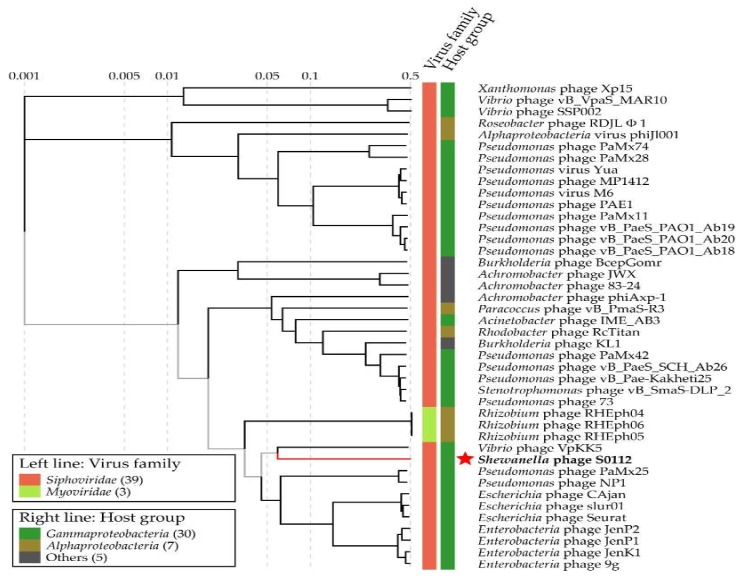
The viral proteomic tree including phage S0112 and other closest relative phages constructed using the ViPTree. The red star represents the *Shewanella* phage S0112.

We used the gene-encoding DNA polymerase subunit B, which has been widely used as a marker gene to study phage diversity to determine the phylogeny of phage S0112. Here, the phylogenetic analysis based on phage DNA polymerase subunit B gene and the viral proteomic tree constructed by the ViPTree both indicated that the closest relative of phage S0112 was *Vibrio* phage VpKK5 (Figure 7 and Figure 8), while, in the whole genome level, phage S0112 and VpKK5 share very low similarity with each other (Figure S1). Phylogenetic analysis combined with morphological assessment indicated that phage S0112 could be a representative of a novel genus belonging to the family *Siphoviridae*.

### 3.7. Environmental Distribution

By using the phage genome of S0112 as queries to search against the IMG/VR database, which is the largest available database of viral genomics, with parameters described elsewhere (blastn -db ~/IMG/VR -query queryname -outfmt “6 std qlen slen” -evalue 1e-5 -max_target_seqs 1000) [61], we did not find any homologous contig and genome, further indicating the novelty of phage S0112. Reciprocal best-hit BLAST showed that the phage proteins have very low similarity to those in both POV and GOS databases, revealing that this kind of phage may rarely exists in other environments beside sediment (Figure S2).

## 4. Conclusions

The benthic phage S0112 infecting *Shewanella indica* KJW27 is a novel lytic phage that was isolated from coastal sediments and can be considered a representative of a novel genus under the family *Siphoviridae*. Fifty-nine percent of its ORFs are functionally unknown. Its strong replication ability, as reflected by the high burst size (i.e., 1170), and its relatively longer latency might be adaptive survival strategies for coastal sediment environments. Several auxiliary metabolic genes were observed in the phage genome, i.e., the pyrophosphohydrolase (MazG) gene and a gene cluster that may be involved in 7-cyano-7-deazaguanine (preQ_0_) biosynthesis. To the best of our knowledge, this study represents the first report of a benthic phage infecting *Shewanella* and sheds light on the phage-host interactions in coastal sediments. As, so far, very few phages have been isolated from coastal sediments, which largely limits our understanding of their biological characteristics, the isolation and characterization of more benthic phages infecting different hosts in coastal sediments are vital for unveiling the phage–host interactions in coastal sediments and their ecological significance, as well as the largely unknown metagenomic dark matters of sediment phages.

## Figures and Tables

**Table 1:** Infection specificities of the phage S0112 against eighteen bacterial strains.

Genus and Species	Strain	Source	Strains Lysed by Phage S0112
*Shewanella indica*	KJW27 ^a^	sediment	+
*Shewanella basaltis*	CJW-54	sediment	-
*Shewanella chilikensis*	JC5	sediment	-
*Shewanella japonica*	KCTC22435	sediment	-
*Shewanella algae*	JCM 21037	seawater	-
*Alginatibacterium sediminis*	ALS 81	sediment	-
*Woeseia oceani*	SDUM189001	sediment	-
*Sediminicola luteus*	SDUM701001	sediment	-
*Kordiimonas sediminis*	N39	sediment	-
*Roseobacter denitrificans*	OCH114	seawater	-
*Marinobacter vinifirmus*	D7035	wastewater	-
*Halomonas denitrificans*	D7027	saline water	-
*Vibrio alginolyticus*	CIP 82.01	seawater	-
*Vibrio neocaledonicus*	NC 470	seawater	-
*Vibrio azureus*	NBRC 104587	seawater	-
*Vibrio harveyi*	NBRC 15634	seawater	-
*Vibrio parahaemolyticus*	NBRC 12711	seawater	-
*Vibrio campbellii*	CAIM 519	seawater	-

+, Lysed; -, not lysed. **^a^** Host strain used in this study.

**Table 2:** Stability of *Shewanella* phage S0112 treated with various physical and chemical agents.

Phage Name	−20 °C	15 °C	20 °C	28 °C	30 °C	37 °C	40 °C	50 °C	60 °C	95 °C	100 °C	pH 2	pH 4	pH 10	pH 12	Osmotic Shock	0.1% CTAB	0.09% SDS	0.1% Sarkosyl	63% Ethanol	90% Acetone	50% DMSO	Chloroform
phage S0112	95	1.3	4.5	99.2	100	86.7	72.9	1.4	0.04	0	0	0	6.8	82.1	0.3	30.4	52.5	0	12.7	1.8	0.4	30.9	91.9

Comparison of the effects of different physical and chemical agents on the survival of phages S0112. Percent of surviving phages under various conditions is shown.

**Table 3:** The genome information of ten *Shewanella* phages.

*Shewanella* Phages ^a^	Host Strains	Family	Size (Kb)	GC%	Protein	No. of tRNAs	Isolation Source
S0112	*Shewanella indica*	*Siphoviridae*	62.286	44.7	102	0	Coastal sediment
Spp001	*S. putrefaciens*	*Siphoviridae*	54.789	49.4	67	0	Sewage
3/49	*S. baltica*	*Siphoviridae*	40.161	42	70	0	Baltic Sea ice
1/44	*S. frigidimarina*	*Siphoviridae*	49.64	39.8	75	0	Baltic Sea ice
SppYZU05	*S. putrefaciens*	*Siphoviridae*	54.319	50.63	65	0	Waste effluents
1/41	*S. baltica*	*Myoviridae*	43.51	42.7	69	0	Baltic Sea ice
SFCi1	*S. fidelis*	*Myoviridae*	42.279	59.1	40	0	Seawater
SppYZU01	*S. baltica*	*Myoviridae*	43.567	55.72	49	0	Waste effluents
1/4	*S. frigidimarina*	*Myoviridae*	133.824	36.9	235	3	Baltic Sea ice
1/40	*S. baltica*	*Myoviridae*	139.004	36.9	236	3	Baltic Sea ice

^a^ Phage S0112 was obtained in the present study. Data for the other nine phages are from the references [12,13,14,15].

**Table 4:** Mass spectrometry analysis of phage S0112 virion.

Detected Protein	Predicted Function	Molecular Mass (kDa)	Number of Peptides	Sequence Coverage (%)	Protein Score
S0112_001	Uncharacterized protein	11.2	3	33.7	175
S0112_065	Uncharacterized protein	52.6	17	46.7	3837
S0112_066	Head-tail connector protein	19.6	7	49.7	1873
S0112_068	Uncharacterized protein	16.9	5	30.1	575
S0112_069	Uncharacterized protein	43.1	4	9.4	220
S0112_071	Major capsid protein	37.3	13	42.1	9723
S0112_074	Tail completion protein	13.7	4	43.7	418
S0112_075	Tail terminator protein	16.1	6	44.3	694
S0112_076	Major capsid protein	54.1	17	58.7	15,158
S0112_079	Tail length tape measure protein	99	24	31.4	5007
S0112_080	Uncharacterized protein	38.4	5	27.1	1859
S0112_081	Virion structural protein	30.8	7	30.8	709
S0112_082	Tail assembly structural protein	62.2	8	27.1	1781
S0112_083	Minor tail protein	30.8	8	37.7	1024
S0112_086	Tail assembly structural protein	83.3	12	23.9	1972
S0112_087	Minor tail protein	14.9	4	45.5	696

## Supplementary Materials

The following are available online at https://www.mdpi.com/1999-4915/11/11/1081/s1. Table S1: Summary of latent period and burst size of sixty-eight phages belonging to the order *Caudovirales*. Table S2: Effects of rifampicin on the phage progeny production. Table S3: The *Shewanella* sp. phage S0112 genome annotations (MK675901). Figure S1: Mauve alignment of phage S0112 genome against phage VpKK5 genome. Figure S2: Prevalence of phage S0112-like ORFs in Pacific Ocean Virome (POV) and Global Ocean Survey (GOS) metagenomic datasets, using the reciprocal best-BLAST strategy.
