# Supplementary material for: A Novel Benthic Phage Infecting Shewanella with Strong Replication Ability
Source: Viruses. 2019 Nov 19;11(11):1081. doi: 10.3390/v11111081 (PMC6893657; doi:10.3390/v11111081)
Supplement: Supplementary file 1 [file viruses-11-01081-s001.pdf]

A Novel Benthic Phage Infecting *Shewanella* With Strong Replication Ability

Zengmeng Wang [1,2], Jiulong Zhao [1,2], Long Wang [1], Chengcheng Li [1,2], Jianhui Liu[3], Lihua Zhang[3], and Yongyu Zhang [1,2]*

Table S1. Summary of latent period and burst size of sixty-eight phages belonging to the order *Caudovirales.*

Phage	Classification	Host	Latent period	Burst size	Reference
vB_RpoMi-Mini	*Microviridae*	*Roseobacter pomeroyi* DSS-3	3 h	8	Zhan and colleagues (2019) [1]
H188	*Myoviridae*	*Vibrio kanaloae*	96 min	3	Li and colleagues (2016) [2]
VVP1	*Myoviridae*	*Vibrio parahaemolyticus*	20-30 min	-	Stalin and colleagues (2016) [3]
PH357	*Myoviridae*	*Pseudoalteromonas lipolytica*	20 min	85	Gong and colleagues (2017) [4]
PH101	*Myoviridae*	*Pseudoalteromonas marina*	20 min	31.6	Wang and colleagues (2015) [5]
IHQ1	*Myoviridae*	*Aeromonas punctata*	24 min	626	Ul Haq and colleagues (2012) [6]
vB-GEC_Ab-M-G7	*Myoviridae*	*Acinetobacter baumannii*	20 min	120	Kusradze and colleagues (2015) [7]
JG024	*Myoviridae*	*Pseudomonas aeruginosa*	50 min	180	Garbe and colleagues (2010) [8]
AP22	*Myoviridae*	*Acinetobacter baumannii*	5 min	240	Lin and colleagues (2010) [9]
vB_PaeM_SCUT-S1	*Myoviridae*	*Pseudomonas aeruginosa*	40 min	134	Guo and colleagues (2019) [10]
vB_PaeM_SCUT-S2	*Myoviridae*	*Pseudomonas aeruginosa*	25 min	40	Guo and colleagues (2019) [10]
VR5	*Myoviridae*	*Escherichia coli* B^E	31 min	50	Kaliniene and colleagues (2010) [11]
VR7	*Myoviridae*	*Escherichia coli* B^E	36 min	70	Kaliniene and colleagues (2010) [11]
VR20	*Myoviridae*	*Escherichia coli* B^E	32 min	48	Kaliniene and colleagues (2010) [11]
KTN6	*Myoviridae*	*Pseudomonas aeruginosa*	35 min	64	Kaliniene and colleagues (2010) [12]
KT28	*Myoviridae*	*Pseudomonas aeruginosa*	36 min	96	Danis-Wlodarczyk and colleagues (2015) [12]
Abp53	*Myoviridae*	*Acinetobacter baumannii*	10 min	150	Lee and colleagues (2011) [13]
RD-1419W1-01	*Podoviridae*	*Roseobacter denitrificans* OCh114	60 min	27	Li and colleagues (2016) [14]

RD-1410Ws-07	*Podoviridae*	*Roseobacter denitrificans* OCh114	<60 min	341	Li and colleagues (2016) [14]
DS-1410Ws-06	*Podoviridae*	*Dinoroseobacter shibae* DFL12	2 h	298	Li and colleagues (2016) [14]
DSS3Φ2	*Podoviridae*	*Silicibacter pomeroyi* DSS-3	3 h	350	Zhao and colleagues (2009) [15]
EE36Φ1	*Podoviridae*	*Sulfitobacter* sp. EE-36	2 h	1500	Zhao and colleagues (2009) [15]
RLP1	*Podoviridae*	*Roseovarius* sp. 217	4 h	100	Chan and colleagues (2014) [16]
RPP1	*Podoviridae*	*Roseobacter nubinhibens*	6 h	10	Chan and colleagues (2014) [16]
vB_ValP_IME271	*Podoviridae*	*Vibrio alginolyticus*	90 min	40	Li and colleagues (2019) [17]
PH1	*Podoviridae*	*Pseudoalteromonas* sp.	30 min	51.3	Liu and colleagues (2017) [18]
phiAxp-3	*Podoviridae*	*Achromobacter xylosoxidans*	80 min	9000	Ma and colleagues (2016) [19]
JWAlpha	*Podoviridae*	*Achromobacter xylosoxidans*	1.5-2 h	180	Wittmann and colleagues (2016) [20]
JWDelta	*Podoviridae*	*Achromobacter xylosoxidans*	1.5-2 h	180	Katharios and colleagues (2017) [21]
vB_VspP_pVa5	*Podoviridae*	*Vibrio splendidus*	30 min	24	Katharios and colleagues (2017) [21]
VMY22	*Podoviridae*	*Bacillus cereus* MYB41-22	70 min	78	Ji and colleagues (2015) [22]
S-B64	*Podoviridae*	*Synechococcus* WH8102	3 h	23	You and colleagues (2019) [23]
Vp670	*Podoviridae*	*Vibrio alginolyticus*	30 min	84	Luo and colleagues (2018) [24]
Φa318	*Podoviridae*	*Vibrio alginolyticus*	15 min	72	Lin and colleagues (2012) [25]
Abp1	*Podoviridae*	*Acinetobacter baumannii*	10 min	350	Huang and colleagues (2013) [26]
ΦIBB-PF7A	*Podoviridae*	*Pseudomonas fluorescens*	15 min	153	Sillankorva and colleagues (2008) [27]
PSa3	*Podoviridae*	*Staphylococcus aureus*	40 min	19	Kraushaar and colleagues (2013) [28]
BVPaP-3	*Podoviridae*	*Pseudomonas aeruginosa*	20 min	44	Ahiwale and colleagues (2012) [29]
KP34	*Podoviridae*	*Klebsiella pneumoniae*	15 min	40-50	Drulis-Kawa and colleagues (2011) [30]
vB_Klp_5	*Podoviridae*	*Klebsiella pneumoniae*	25 min	90	Karumidze and colleagues (2013) [31]
ΦAB2	*Podoviridae*	*Acinetobacter baumannii*	10 min	200	Lin and colleagues (2010) [9]
RDJL Φ1	*Siphoviridae*	*Roseobacter denitrificans* OCh114	80 min	203	Zhang and colleagues (2009) [32]
vB_DshS-R5C	*Siphoviridae*	*Dinoroseobacter shibae* DFL12	1.5–2 h	65	Yang and colleagues (2017) [33]
BS5	*Siphoviridae*	*Pseudoalteromonas atlantica*	80 min	95	Meng and colleagues (2017) [34]

B8b	*Siphoviridae*	*Pseudoalteromonas* sp. QC-44	70 min	172	Lara and colleagues (2015) [35]
P1	*Siphoviridae*	*Lactobacillus plantarum* IMAU10120	45 min	133	Chen and colleagues (2016) [36]
PB15	*Siphoviridae*	*Alteromonas gracilis*	16 min	60	Gao and colleagues (2017) [37]
B23	*Siphoviridae*	*Marinobacter salarius* R9SW1	90 min	19.3	Zhu and colleagues (2018) [38]
vB_EliS-R6L	*Siphoviridae*	*Erythrobacter litoralis* DSM 8509	2.7 h	86	Lu and colleagues (2017) [39]
AhSzq-1	*Siphoviridae*	*Aeromonas hydrophila*	50 min	45	Yuan and colleagues (2018) [40]
AhSzw-1	*Siphoviridae*	*Aeromonas hydrophila*	60 min	45	Yuan and colleagues (2018) [40]
ΦBM	*Siphoviridae*	*Bacillus megaterium*	40 min	65	Othman and colleagues (2015) [41]
GE1	*Siphoviridae*	*Lactococcus garvieae*	31 min	139	Eraclio and colleagues (2015) [42]
vB_Klox_2	*Siphoviridae*	*Klebsiella oxytoca*	20 min	346	Karumidze and colleagues (2013) [31]
ΦIF3	*Siphoviridae*	*Serratia marcescens*	50 min	100	Petty and colleagues (2006) [43]
vB_SenS-Ent1	*Siphoviridae*	*Salmonella enterica*	25 min	35	Turner and colleagues [44]
SFci1	*Myoviridae*	*Shewanella. fidelis*	60 min	62	Leigh and colleagues [45]
Spp001	-	*Shewanella.putrefaciens* Sp225	40 min	-	Han and colleagues [46]
SppYZU01	*Myoviridae*	*Shewanella baltica* SYZU01	22.3 min	55±3	Yang and colleagues [47]
SppYZU02	*Siphoviridae*	*Shewanella putrefaciens* SYZU02	12.7 min	81±16	Yang and colleagues [47]
SppYZU03	*Myoviridae*	*Shewanella baltica* SYZU03	27.1 min	39±5	Yang and colleagues [47]
SppYZU04	*Siphoviridae*	*Shewanella putrefaciens* SYZU04	42.7 min	37±4	Yang and colleagues [47]
SppYZU05	*Siphoviridae*	*Shewanella putrefaciens* SYZU05	33.7 min	68±5	Yang and colleagues [47]
SppYZU06	*Myoviridae*	*Shewanella putrefaciens* SYZU06	35.3 min	45±6	Yang and colleagues [47]
SppYZU07	*Myoviridae*	*Shewanella putrefaciens* SYZU07	53.7 min	33±9	Yang and colleagues [47]
SppYZU08	*Myoviridae*	*Shewanella baltica* SYZU08	45 min	23±3	Yang and colleagues [47]
SppYZU09	*Siphoviridae*	*Shewanella baltica* SYZU09	28 min	25±5	Yang and colleagues [47]
SppYZU10	*Myoviridae*	*Shewanella putrefaciens* SYZU10	42.3 min	20±1	Yang and colleagues [47]

Table S2. Effects of rifampicin on the phage progeny production.

Phage	Rifampicin (μg/mL)	Number of Plaques × 10^8
phage S0112	0	385 ± 40
	5	0
	10	0
	20	0

Table S3. The *Shewanella* sp. phage S0112 genome annotations (MK675901).

ORF No.	Strand	Start	End	Best Homologs	Accession	E-Value (<10^{-3})	aa Identity	Best Domain Hits (E-value < 10^{-3})/Accession	Putative Function
1	-	159	473	*Vibrio* phage 1.117.O._10N.261.45.E9	AUR88738.1	3.00E-25	63.64%		Hypothetical protein NVP1117O_60
2	-	556	1155	*Burkholderia* phage BcepGomr	YP_001210260.1	9.00E-07	39.76%		Hypothetical protein BcepGomr*gp*40
3	-	1271	1543	None	n/a	n/a	n/a		Hypothetical protein
4	-	1598	1879	*Xanthomonas* phage XAJ2	AMW36195.1	0.11	46.15%		Hypothetical protein
5	-	1866	3554	*Vibrio* phage VpKK5	YP_009126858.1	0	47.49%	SSL2/COG1061	DNA/RNA helicase
6	-	3551	4567	*Pantoea* phage vB_PagS_Vid5	AVJ51801.1	5.00E-93	46.00%		Exonuclease
7	-	4641	5240	*Vibrio* phage vB_VhaS-tm	ANO57479.1	2.00E-33	46.32%		Multimodular transpeptidase-transglycosylase
8	-	5283	5624	None	n/a	n/a	n/a		Hypothetical protein
9	-	5629	6357	*Vibrio* phage VpKK5	YP_009126576.1	5.00E-91	56.78%	P-loop_NTPase/cl21455	ATPase
10	-	6357	6593	None	n/a	n/a	n/a		Hypothetical protein
11	-	6599	7453	*Vibrio* phage 1.117.O._10N.261.45.E9	AUR88750.1	2.00E-136	94%	ClpX/cl26528	P-loop containing nucleoside triphosphate hydrolase

12	-	7532	7972	*Vibrio* phage 1.117.O._10N.261.45.E9	AUR88751.1	2.00E-12	34.65%		Hypothetical protein NVP1117O_73
13	-	7987	8208	None	n/a	n/a	n/a		Hypothetical protein
14	-	8211	8603	None	n/a	n/a	n/a		Hypothetical protein
15	-	8609	9022	None	n/a	n/a	n/a		Hypothetical protein
16	-	9019	9336	None	n/a	n/a	n/a		Hypothetical protein
17	+	9486	9695	*Microcystis* phage Me-ZS1	AZF88181.1	1.00E-04	55.56%		DNA-binding domain protein
18	+	9705	12230	*Vibrio* phage VpKK5	YP_009126572.1	2.00E-112	42.79%	COG4983	Primase/helicase
19	+	12227	12406	None	n/a	n/a	n/a		Hypothetical protein
20	+	12418	12726	*Vibrio* phage 1.205.O._10N.222.51.A7	AUR95312.1	3.00E-10	41.10%		Hypothetical protein NVP1205O_37
21	+	12816	13160	*Acinetobacter* phage Acj9	YP_004010343.1	7.00E-05	27.78%		Hypothetical protein Acj9p206
22	+	13136	13399	None	n/a	n/a	n/a		Hypothetical protein
23	+	13399	13962	*Shewanella* sp. phage 1/4	YP_009100322.1	9.00E-36	39.43%	DNA_processg_A/cl22881	DprA-like protein
24	+	14011	14202	*Pseudoalteromonas* phage PH1	YP_009321573.1	8.00E-05	39.02%		Hypothetical protein
25	-	14199	14399	None	n/a	n/a	n/a		Hypothetical protein
26	+	14398	14514	None	n/a	n/a	n/a		Hypothetical protein
27	+	14501	14728	None	n/a	n/a	n/a		Hypothetical protein
28	+	14715	14987	*Pseudoalteromonas gelatinilytica*	WP_063705229.1	1.00E-04	36.92%		Hypothetical protein
29	+	14974	15429	None	n/a	n/a	n/a		Hypothetical protein
30	+	15426	15614	None	n/a	n/a	n/a		Hypothetical protein
31	+	15646	15861	*Burkholderia ubonensis*	WP_059928282.1	5.00E-08	37.70%		Hypothetical protein
32	+	15969	16187	*Vibrio* phage vB_VmeM-32	ALY07163.1	1.00E-04	33.33%		Hypothetical protein VmeM32_00177
33	+	16239	16565	*Pseudomonas* phage PaBG	YP_008433597.1	5.00E-11	35.92%		Hypothetical protein PaBG_00267
34	+	16578	17132	*Siphoviridae* sp.	AXQ65838.1	2.00E-05	28.57%		Peptidase C39 family protein
35	+	17129	17698	*Vibrio* virus Thalassa	AUG85379.1	2.00E-11	37.86%	57B/PHA02574	Hypothetical protein THALASSA_200

36	+	18129	18569	*Pantoea* phage vB_PagS_Vid5	AVJ51809.1	1.00E-14	31.72%		Endonuclease
37	+	18745	19440	*Burkholderia* phage BcepGomr	YP_001210278.1	6.00E-35	37.66%	DUF2786/pfam10979	Hypothetical protein BcepGomr*gp*58
38	+	19449	19559	None	n/a	n/a	n/a		Hypothetical protein
39	+	19744	19917	None	n/a	n/a	n/a		Hypothetical protein
40	+	19965	20195	None	n/a	n/a	n/a		Hypothetical protein
41	+	20192	20377	None	n/a	n/a	n/a		Hypothetical protein
42	+	20374	20571	None	n/a	n/a	n/a		Hypothetical protein
43	-	20594	20794	None	n/a	n/a	n/a		Hypothetical protein
44	+	21003	21344	*Microcystis* phage Me-ZS1	AZF88115.1	7.00E-11	51.61%		Hypothetical protein
45	+	21649	21807	None	n/a	n/a	n/a		Hypothetical protein
46	-	21810	21914	None	n/a	n/a	n/a		Hypothetical protein
47	+	21913	22239	None	n/a	n/a	n/a		Hypothetical protein
48	-	22302	22463	None	n/a	n/a	n/a		Hypothetical protein
49	+	22725	23252	None	n/a	n/a	n/a		Hypothetical protein
50	+	23254	23499	*Vibrio* phage douglas 12A4	YP_007877480.1	5.00E-18	48.15%		Hypothetical protein VPAG_00055
51	+	23496	23825	*Shewanella* sp. Shew256	WP_088213025.1	2.00E-21	45.37%		Hypothetical protein
52	+	23827	24162	None	n/a	n/a	n/a		Hypothetical protein
53	+	24152	24709	*Escherichia* phage LAMP	AUM57625.1	2.00E-04	39.44%		Hypothetical protein phiL_054
54	+	24713	24862	None	n/a	n/a	n/a		Hypothetical protein
55	+	24924	25403	None	n/a	n/a	n/a		Hypothetical protein
56	+	25422	25877	None	n/a	n/a	n/a		Hypothetical protein
57	+	25903	26082	None	n/a	n/a	n/a		Hypothetical protein
58	+	26082	26537	None	n/a	n/a	n/a		Hypothetical protein
59	+	26534	26722	None	n/a	n/a	n/a		Hypothetical protein
60	+	26905	27582	*Pseudoalteromonas piratica*	WP_038640832.1	5.00E-35	39.15%	Abhydrolase_5/pfam12695	*Alpha/beta* hydrolase
61	+	27548	27799	None	n/a	n/a	n/a		Hypothetical protein
62	+	27807	28289	*Alteromonas* phage ZP6	AZS06533.1	1.00E-18	44.96%		Putative endolysin
63	+	28393	29145	*Bordetella* phage MW2	APL99191.1	7.00E-05	30.83%		Terminase small subunit
64	+	29142	30800	*Fodinicurvata sediminis*	WP_081649884.1	3.00E-161	49.29%	COG5410	Terminase large subunit

65	+	30862	32283	*Roseobacter* phage RDJL Φ 1	YP_004421835.1	7.00E-141	50.84%	Phage_portal/cl19194	Hypothetical protein RDJLΦ1_*gp*67
66	+	32283	32834	Uncultured Mediterranean phage uvMED	BAR14449.1	1.00E-10	30.00%		Head-tail connector protein
67	+	32834	33202	*Pseudomonas* phage KPP23	BAO53109.1	2.00E-16	42.15%		Hypothetical protein KPP23_082
68	+	33211	33651	*Faecalibacterium prausnitzii*	WP_097778004.1	3.00E-04	28.08%		Hypothetical protein
69	+	33644	34831	*Roseobacter* phage RDJL Φ 1	YP_004421840.1	7.00E-23	32.69%		Hypothetical protein RDJLΦ1_*gp*72
70	+	34843	35235	*Pseudomonas* phage KPP23	BAO53113.1	1.00E-5	33.3%		Hypothetical protein
71	+	35246	36253	*Vibrio* phage 1.012.O._10N.261.48.C12	AUR81674.1	8.00E-94	47.35%	Phage_cap_E/pfam03864	Major capsid protein
72	+	36312	36656	None	n/a	n/a	n/a		Hypothetical protein
73	+	36647	37228	*Shewanella* sp. phage 1/4	YP_009100432.1	7.00E-52	49.08%		Hypothetical protein S14_128
74	+	37221	37601	*Pseudomonas* phage vB_PaeS_SCUT-S3	AZF90025.1	1.00E-07	32.20%		Tail completion protein
75	+	37621	38043	*Pseudomonas* phage JG054	ARB11157.1	7.00E-08	27.34%	DUF4128/pfam13554	Tail terminator protein
76	+	38055	39584	*Roseobacter* phage RDJL Φ 1	YP_004421846.1	3.00E-179	52.94%		Major capsid protein
77	+	39614	40036	*Roseobacter* phage RDJL Φ 1	YP_004421847.1	2.00E-30	38.89%		Hypothetical protein RDJLΦ1_*gp*79
78	+	40168	40416	*Achromobacter* phage phiAxp-1	YP_009220352.1	2.00E-08	44.44%		Tail chaperonin
79	+	40426	43230	*Bordetella* phage MW2	APL99211.1	2.00E-95	32.46%	COG5281	Tail length tape measure protein
80	+	43232	44287	*Pseudomonas aeruginosa*	WP_058198295.1	3.00E-23	34.25%		Hypothetical protein
81	+	44289	45236	*Pseudomonas* phage AAT-1	AME18051.1	1.00E-09	24.70%		Virion structural protein
82	+	45236	46882	*Pseudomonas* phage MP1412	YP_006561079.1	3.00E-75	31.56%		Tail assembly structural protein
83	+	46885	47715	*Pseudomonas* phage LKO4	AGI11321.1	2.00E-65	39.13%	phg_TIGR02218/TIGR02218	Minor tail protein
84	+	47726	47950	*Pseudomonas* phage MP1412	YP_006561081.1	6.00E-16	56.94%		Tail assembly structural protein
85	+	47950	48147	*Vibrio* phage VpKK5	YP_009126598.1	3.00E-09	41.94%		Tail assembly protein

86	+	48134	50416	*Pseudomonas* phage MP1412	YP_006561083.1	9.00E-169	41.89%	Phage-tail_3/pfam13550	Tail assembly structural protein
87	+	50419	50850	*Gordonia* phage Bizzy	AYR02676.1	3.00E-19	50.00%		Minor tail protein
88	+	50847	53066	*Rhizobium album*	PWE57125.1	7.50E-7	31.3%		Hypothetical protein
89	+	53145	53582	None	n/a	n/a	n/a		Hypothetical protein
90	+	53603	53995	None	n/a	n/a	n/a		Hypothetical protein
91	+	54005	54271	*Alphaproteobacteria* virus phiJl001	YP_223928.1	3.00E-16	46.25%		Hypothetical protein *gp*04
92	-	54268	56259	*Paracoccus* phage vB_PmaS-R3	YP_009126392.1	0	44.53%	DnaQ_like_exo/cl10012	DNA polymerase I
93	-	56262	57212	*Vibrio* phage VpKK5	YP_009126592.1	2.00E-107	49.68%	beta_clamp/cl21574	DNA polymerase subunit B
94	-	57187	57459	*Pseudomonas* phage KTN4	ANM44782.1	1.00E-05	38.96%		Hypothetical protein KTN4_024
95	-	57459	58376	*Vibrio* phage VpKK5	YP_009126591.1	6.00E-117	59.01%		Queuosine tRNA-ribosyltransferase
96	-	58376	58828	*Klebsiella* phage May	AUG88010.1	3.00E-26	39.68%		Hypothetical protein CPT_May_096
97	-	58904	59488	*Pantoea* phage vB_PagS_Vid5	AVJ51790.1	8.00E-88	65.22%	folE/PRK09347	GTP cyclohydrolase type I
98	-	59501	59962	*Enterobacteria* phage 9g	YP_009032328.1	1.00E-43	44.89%	PTPS/pfam01242	6-carboxy-5,6,7,8-tetrahydropterin synthase
99	-	59962	60525	*Acinetobacter baumannii* 1295743	EXB03771.1	4.00E-11	42.22%		mazG nucleotide pyrophosphohydrolase domain protein
100	-	60525	61271	*Vibrio* phage VpKK5	YP_009126588.1	2.00E-103	64.71%	QueC/pfam06508	7-cyano-7-deazaguanine synthase
101	-	61279	62013	*Vibrio* phage VpKK5	YP_009126587.1	8.00E-61	46.12%	NrdG/COG0602	Orgamic radical activating enzyme
102	-	62134	62286	None	n/a	n/a	n/a		Hypothetical protein

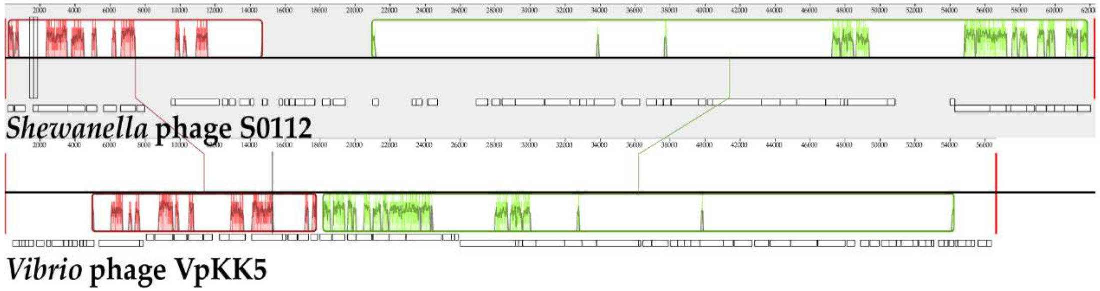

Figure S1. Mauve alignment of phage S0112 genome against phage VpKK5 genome. Colored boxes indicate homologous DNA regions between genomes without genomic rearrangements. Genomic similarity is represented by the height of the bars, which correspond to the average level of conservation in that region of the genome sequence. Completely white regions represent fragments that were not aligned or contained sequence elements specific to a particular genome.

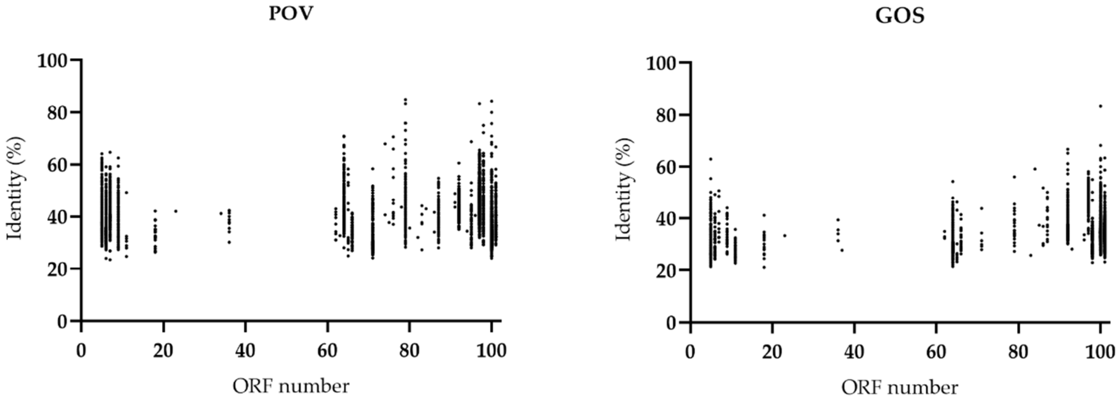

Figure S2. Prevalence of phage S0112-like ORFs in Pacific Ocean Virome (POV) and Global Ocean Survey (GOS) metagenomic datasets using the reciprocal best-BLAST strategy.

References

1. Zhan, Y.; Chen, F. The smallest ssDNA phage infecting a marine bacterium. *Environ. Microbiol.* **2019**, *21*, 1916-1928.
2. Li, Y.; Wang, M.; Shao, H.; Liu, Q.; Jiang, Y.; Song, X.; Wang, D.; Ma, Y. Complete genomic sequence of bacteriophage H188: A novel *Vibrio kanaloae* phage isolated from Yellow Sea. *Curr. Microbiol.* **2016**, *72*, 628-633.
3. Stalin, N.; Srinivasan, P. Characterization of *Vibrio parahaemolyticus* and its specific phage from shrimp pond in Palk Strait, South East coast of India. *Biologicals* **2016**, *44*, 526-533.
4. Gong, Z.; Wang, M.; Yang, Q.; Li, Z.; Xia, J.; Gao, Y.; Jiang, Y.; Meng, X.; Liu, Z.; Yang, D.; et al. Isolation and complete genome sequence of a novel *Pseudoalteromonas* phage PH357 from the Yangtze River Estuary. *Curr. Microbiol.* **2017**, *74*, 832-839.
5. Wang, D.; Sun, M.; Shao, H.; Li, Y.; Meng, X.; Liu, Z.; Wang, M. Characterization and genome sequencing of a novel bacteriophage PH101 infecting *Pseudoalteromonas marina* BH101 from the Yellow Sea of China. *Curr. Microbiol.* **2015**, *71*, 594-600.
6. Ul Haq, I.; Chaudhry, W. N.; Andleeb, S.; Qadri, I. Isolation and partial characterization of a virulent bacteriophage IHQ1 specific for *Aeromonas punctata* from stream water. *Microb. Ecol.* **2012**, *63*, 954-963.
7. Kusradze, I.; Karumidze, N.; Rigvava, S.; Dvalidze, T.; Katsitadze, M.; Amiranashvili, I.; Goderdzishvili, M. Characterization and testing the efficiency of *Acinetobacter baumannii* phage vB-GEC_Ab-M-G7 as an antibacterial agent. *Front. Microbiol.* **2016**, *7*, 1590.
8. Garbe, J.; Wesche, A.; Bunk, B.; Kazmierczak, M.; Selezska, K.; Rohde, C.; Sikorski, J.; Rohde, M.; Dieter Jahn, D.; Schobert, M. Characterization of JG024, a *Pseudomonas aeruginosa* PB1-like broad host range phage under simulated infection conditions. *BMC Microbiol.* **2010**, *10*, 301.
9. Lin, N.T.; Chiou, P.Y.; Chang, K.C.; Chen, L.K.; Lai, M.J. Isolation and characterization of phi AB2: a novel bacteriophage of *Acinetobacter baumannii*. *Res. Microbiol.* **2010**, *161*, 308-314.
10. Guo, Y.; Chen, P.; Lin, Z.; Wang, T. Characterization of two *Pseudomonas aeruginosa* viruses vB_PaeM_SCUT-S1 and vB_PaeM_SCUT-S2. *Viruses* **2019**, *11*, 318.
11. Kaliniene, L.; Klausa, V.; Truncaite, L. Low-temperature T4-like coliphages vB_EcoM-VR5, vB_EcoM-VR7 and vB_EcoM-VR20. *Arch. Virol.* **2010**, *155*, 871-880.
12. Danis-Wlodarczyk, K.; Olszak, T.; Arabski, M.; Wasik, S.; Majkowska-Skrobek, G.; Augustyniak, D.; Gula, G.; Briers, Y.; Jang, H. B.; Vandenheuvel, D.; et al. Characterization of the newly isolated lytic bacteriophages KTN6 and KT28 and their efficacy against *Pseudomonas aeruginosa* biofilm. *PLoS One* **2015**, *10*, e0127603.
13. Lee, C.N.; Tseng, T.T.; Lin, J.W.; Fu, Y.C.; Weng, S.F.; Tseng, Y.H. Lytic myophage Abp53 encodes several proteins similar to those encoded by host *Acinetobacter baumannii* and phage phiKO2. *Appl. Environ. Microbiol.* **2011**, *77*, 6755-6762.
14. Li, B.; Zhang. S.; Long. L.; Huang, S. Characterization and complete genome sequences of three N4-Like *Roseobacter* phages isolated from the South China Sea. *Curr. Microbiol.* **2016**, *73*, 409-418.

15. Zhao, Y.; Wang, K.; Jiao, N.; Chen, F. Genome sequences of two novel phages infecting marine roseobacters. *Environ. Microbiol.* **2009**, *11*, 2055-2064.
16. Chan, J.Z.; Millard, A.D.; Mann, N.H.; Schafer, H. Comparative genomics defines the core genome of the growing N4-like phage genus and identifies N4-like Roseophage specific genes. *Front. Microbiol.* **2014**, *5*, 506.
17. Li, F.; Xing, S.; Fu, K.; Zhao, S.; Liu, J.; Tong, Y.; Zhou, L. Genomic and biological characterization of the *Vibrio alginolyticus*-infecting "*Podoviridae*" bacteriophage, vB_ValP_IME271. *Virus Genes* **2019**, *55*, 218-226.
18. Liu, Z.; Wang, M.; Meng, X.; Li, Y.; Wang, D.; Jiang, Y.; Shao, H.; Zhang, Y. Isolation and genome sequencing of a novel *Pseudoalteromonas* phage PH1. *Curr. Microbiol.* **2017**, *74*, 212-218.
19. Ma, Y.; Li, E.; Qi, Z.; Li, H.; Wei, X.; Lin, W.; Zhao, R.; Jiang, A.; Yang, H.; Yin, Z.; et al. Isolation and molecular characterisation of *Achromobacter* phage phiAxp-3, an N4-like bacteriophage. *Sci. Rep.* **2016**, *6*, 24776.
20. Wittmann, J.; Dreiseikelmann, B.; Rohde, M.; Meier-Kolthoff, J.P.; Bunk, B.; Rohde, C. First genome sequences of *Achromobacter* phages reveal new members of the N4 family. *J. Virol.* **2014**, *11*, 14.
21. Katharios, P.; Kalatzis, P.G.; Kokkari, C.; Sarropoulou, E.; Middelboe, M. Isolation and characterization of a N4-like lytic bacteriophage infecting *Vibrio splendidus*, a pathogen of fish and bivalves. *PLoS One* **2017**, *12*, e0190083.
22. Ji, X.; Zhang, C.; Fang, Y.; Zhang, Q.; Lin, L.; Tang, B.; Wei, Y. Isolation and characterization of glacier VMY22, a novel lytic cold-active bacteriophage of *Bacillus cereus*. *Virol. Sin.* **2015**, *30*, 52-58.
23. You, S.; Wang, M.; Jiang, Y.; Jiang, T.; Liu, Y.; Liu, X.; Yang, Q.; Shao, H. The genome sequence of a novel cyanophage S-B64 from the Yellow Sea, China. *Curr. Microbiol.* **2019**, *76*, 681-686.
24. Luo, P.; Yun, L.; Li, Y.; Tian, Y.; Liu, Q.; Huang, W.; Hu, C. Complete genomic sequence of the *Vibrio alginolyticus* bacteriophage Vp670 and characterization of the lysis-related genes, cwlQ and holA. *BMC Genomics* **2018**, *19*, 741.
25. Lin, Y.R.; Chiu, C.W.; Chang, F.Y.; Lin, C.S. Characterization of a new phage, termed varphiA318, which is specific for *Vibrio alginolyticus*. *Arch. Virol.* **2012**, *157*, 917-926.
26. Huang, G.; Le, S.; Peng, Y.; Zhao, Y.; Yin, S.; Zhang, L.; Yao, X.; Tan, Y.; Li, M.; Hu, F. Characterization and genome sequencing of phage Abp1, a new phiKMV-like virus infecting multidrug-resistant *Acinetobacter baumannii*. *Curr. Microbiol.* **2013**, *66*, 535-543.
27. Sillankorva, S.; Neubauer, P.; Azeredo, J., Isolation and characterization of a T7-like lytic phage for *Pseudomonas fluorescens*. *BMC Biotechnol.* **2008**, *8*, 80.
28. Kraushaar, B.; Thanh, M.D.; Hammerl, J.A.; Reetz, J.; Fetsch, A.; Hertwig, S. Isolation and characterization of phages with lytic activity against methicillin-resistant *Staphylococcus aureus* strains belonging to clonal complex 398. *Arch. Virol.* **2013**, *158*, 2341-2350.
29. Ahiwale, S.; Prakash, D.; Gajbhiye, M.; Jagdale, S.; Patil, N.; Kapadnis, B. BVPaP-3, a T7-like lytic phage of *Pseudomonas aeruginosa*: its isolation and characterisation. *Curr.*

Microbiol. **2012**, *64*, 305-311.

30. Drulis-Kawa, Z.; Mackiewicz, P.; Kęsik-Szeloch, A.; Maciaszczyk-Dziubinska, E.; Weber-Dąbrowska, B.; Dorotkiewicz-Jach, A.; Augustyniak, D.; Majkowska-Skrobek, G.; Bocer, T.; Empel, J.; et al. Isolation and characterisation of KP34-a novel ΦKMV-like bacteriophage for *Klebsiella pneumoniae*. *Appl. Environ. Microbiol.* **2011**, *90*, 1333-1345.
31. Karumidze, N.; Kusradze, I.; Rigvava, S.; Goderdzishvili, M.; Rajakumar, K.; Alavidze, Z. Isolation and characterisation of lytic bacteriophages of *Klebsiella pneumoniae* and *Klebsiella oxytoca*. *Curr*. *Microbiol*. **2013**, *66*, 251-258.
32. Zhang, Y.; Jiao, N. Roseophage RDJLΦ1, infecting the aerobic anoxygenic phototrophic bacterium *Roseobacter denitrificans* OCh114. *Appl. Environ. Microbiol.* **2009**, *75*, 1745-1749.
33. Yang, Y.; Cai, L.; Ma, R.; Xu, Y.; Tong, Y.; Huang, Y.; Jiao, N.; Zhang, R. A novel roseosiphophage isolated from the Oligotrophic South China Sea. *Viruses* **2017**, *9*, 109.
34. Meng, X.; Wang, M.; You, S.; Wang, D.; Li, Y.; Liu, Z.; Gao, Y.; Liu, L.; Zhang, Y.; Yan, Z.; et al. Characterization and complete genome sequence of a novel *Siphoviridae* bacteriophage BS5. *Curr*. *Microbiol*. **2017**, *74*, 815-820.
35. Lara, E.; Holmfeldt, K.; Solonenko, N.; Sa, E.L.; Ignacio-Espinoza, J.C.; Cornejo-Castillo, F.M.; Verberkmoes, N.C.; Vaque, D.; Sullivan, M.B.; Acinas, S.G. Life-style and genome structure of marine *Pseudoalteromonas* siphovirus B8b isolated from the northwestern Mediterranean Sea. *PLoS One* **2015**, *10*, e0114829.
36. Chen, X.; Xi, Y.; Zhang, H.; Wang, Z.; Fan, M.; Liu, Y.; Wu, W. Characterization and adsorption of *Lactobacillus virulent* phage P1. *J. Dairy Sci*. **2016**, *99*, 6995-7001.
37. Gao, Y.; Liu, Q.; Wang, M.; Zhao, G.; Jiang, Y.; Malin, G.; Gong, Z.; Meng, X.; Liu, Z.; Lin, T.; et al. Characterization and genome sequence of marine *Alteromonas gracilis* phage PB15 isolated from the Yellow Sea, China. *Curr. Microbiol*. **2017**, *74*, 821-826.
38. Zhu, M.; Wang, M.; Jiang, Y.; You, S.; Zhao, G.; Liu, Y.; Yang, Q.; Liu, Q.; Liu, Z.; Gong, Z. et al. Isolation and complete genome sequence of a novel marinobacter phage B23. *Curr*. *Microbiol*. **2018**, *75*, 1619-1625.
39. Lu, L.; Cai, L.; Jiao, N,; Zhang, R. Isolation and characterization of the first phage infecting ecologically important marine bacteria *Erythrobacter*. *J*. *Virol*. **2017**, *14*, 104.
40. Yuan, S.; Chen, L.; Liu, Q.; Zhou, Y.; Yang, J.; Deng, D.; Li, H.; Ma, Y. Characterization and genomic analyses of *Aeromonas hydrophila* phages AhSzq-1 and AhSzw-1, isolates representing new species within the T5virus genus. *Arch*. *Virol*. **2018**, *163*, 1985-1988.
41. Othman, B.A.; Askora, A.; Abo-Senna, A.S., Isolation and characterization of a *Siphoviridae* phage infecting *Bacillus megaterium* from a heavily trafficked holy site in Saudi Arabia. *Folia Microbiol*. (*Praha*) **2015**, *60*, 289-295.
42. Eraclio, G.; Tremblay, D.M.; Lacelle-Cote, A.; Labrie, S.J.; Fortina, M.G.; Moineau, S. A virulent phage infecting *Lactococcus garvieae*, with homology to *Lactococcus lactis* phages. *Appl. Environ. Microbiol.* **2015**, *81*, 8358-8365.
43. Petty, N.K.; Foulds, I.J.; Pradel, E.; Ewbank, J.J.; Salmond, G.P. A generalized transducing phage (phiIF3) for the genomically sequenced *Serratia marcescens* strain Db11: a tool for functional genomics of an opportunistic human pathogen. *Microbiology* **2006**, *152*, 1701-1708.

44. Turner, D.; Hezwani, M.; Nelson, S.; Salisbury, V.; Reynolds, D. Characterization of the *Salmonella* bacteriophage vB_SenS-Ent1. *J. Gen. Virol*. **2012,** *93*, 2046-2056.
45. Leigh, B.; Karrer, C.; Cannon, J.P.; Breitbart, M.; Dishaw, L.J. Isolation and characterization of a *Shewanella* phage-host system from the gut of the tunicate, *Ciona intestinalis*. *Viruses* **2017,** *9*, 60.
46. Han, F.; Li, M.; Lin, H.; Wang, J.; Cao, L.; Khan, M.N. The novel *Shewanella putrefaciens*-infecting bacteriophage Spp001: genome sequence and lytic enzymes. *J. Ind. Microbiol. Biotechnol*. **2014**, *41*, 1017-1026.
47. Yang, Z.; Tao, X.; Zhang, H.; Rao, S.; Gao, L.; Pan, Z.; Jiao, X. Isolation and characterization of virulent phages infecting *Shewanella baltica* and *Shewanella putrefaciens*, and their application for biopreservation of chilled channel catfish (*Ictalurus punctatus*). *Int. J. Food Microbiol*. **2019**, *292*, 107-117.

© 2019 by the authors. Submitted for possible open access publication under the terms and conditions of the Creative Commons Attribution (CC BY) license (http://creativecommons.org/licenses/by/4.0/).
